# Development of Next Generation Vaccines against SARS-CoV-2 and Variants of Concern

**DOI:** 10.3390/v15030624

**Published:** 2023-02-24

**Authors:** Abdul Aziz Al-Fattah Yahaya, Kanwal Khalid, Hui Xuan Lim, Chit Laa Poh

**Affiliations:** Centre for Virus and Vaccine Research, School of Medical and Life Sciences, Sunway University, Bandar Sunway, Petaling Jaya 47500, Selangor, Malaysia

**Keywords:** SARS-CoV-2, variants, vaccines

## Abstract

SARS-CoV-2 has caused the COVID-19 pandemic, with over 673 million infections and 6.85 million deaths globally. Novel mRNA and viral-vectored vaccines were developed and licensed for global immunizations under emergency approval. They have demonstrated good safety and high protective efficacy against the SARS-CoV-2 Wuhan strain. However, the emergence of highly infectious and transmissible variants of concern (VOCs) such as Omicron was associated with considerable reductions in the protective efficacy of the current vaccines. The development of next-generation vaccines that could confer broad protection against both the SARS-CoV-2 Wuhan strain and VOCs is urgently needed. A bivalent mRNA vaccine encoding the Spike proteins of both the SARS-CoV-2 Wuhan strain and the Omicron variant has been constructed and approved by the US FDA. However, mRNA vaccines are associated with instability and require an extremely low temperature (−80 °C) for storage and transportation. They also require complex synthesis and multiple chromatographic purifications. Peptide-based next-generation vaccines could be developed by relying on in silico predictions to identify peptides specifying highly conserved B, CD4^+^ and CD8^+^ T cell epitopes to elicit broad and long-lasting immune protection. These epitopes were validated in animal models and in early phase clinical trials to demonstrate immunogenicity and safety. Next-generation peptide vaccine formulations could be developed to incorporate only naked peptides, but they are costly to synthesize and production would generate extensive chemical waste. Continual production of recombinant peptides specifying immunogenic B and T cell epitopes could be achieved in hosts such as *E. coli* or yeast. However, recombinant protein/peptide vaccines require purification before administration. The DNA vaccine might serve as the most effective next-generation vaccine for low-income countries, since it does not require an extremely low temperature for storage or need extensive chromatographic purification. The construction of recombinant plasmids carrying genes specifying highly conserved B and T cell epitopes meant that vaccine candidates representing highly conserved antigenic regions could be rapidly developed. Poor immunogenicity of DNA vaccines could be overcome by the incorporation of chemical or molecular adjuvants and the development of nanoparticles for effective delivery.

## 1. Introduction

Considering the global spread of COVID-19 is due to its high transmissibility, there is an urgent need to rapidly develop safe and effective vaccines to curb the further spread of the virus. In particular, the alarming threat of the COVID-19 pandemic on global healthcare systems and its impact on the economy has necessitated the urgent development of effective vaccines. Thus, vaccines were developed at a rate unparalleled in the history of human vaccinology. Initial vaccine development against SARS-CoV-2 quickly progressed through the preclinical and clinical stages soon after the whole-genome sequence of the SARS-CoV-2 Wuhan strain became available [1]. Accelerated development of SARS-CoV-2 vaccines occurred as a result of collaborations between governments, universities and big pharma [2]. One such example is the Operation Warp Speed development of SARS-CoV-2 vaccine, a public and private initiative started by the United States Congress aimed to speed up the research, development, manufacturing, and distribution of vaccines. Warp-speed vaccine development against SARS-CoV-2 utilized novel and previous unlicensed platforms [3]. mRNA and viral-vectored vaccines were among the first few vaccine candidates, along with the inactivated vaccine (IV), approved for Phase III clinical development and subsequent approvals for emergency use [4].

The purpose of this paper is to review the development of next-generation vaccines against SARS-CoV-2 and the variants of concern (VOCs), with particular emphasis on vaccine platforms such as mRNA and recombinant protein or peptide-based vaccines. The vaccines described in this review are based on the latest research findings available regarding the vaccine platforms that have yet to be clinically applied to protect against SARS-CoV-2 infections. Promising vaccination approaches such as aerosolized adenovirus or AAV-based vaccines, live attenuated vaccines which could be applied intranasally, and novel vaccine platforms such as peptide-based vaccines all fall under this purview.

A search for next-generation vaccines was conducted using Google Scholar and PubMed databases. The following keyword search terms were used; “SARS-CoV-2” OR “severe acute respiratory syndrome coronavirus-2” AND “bivalent vaccine” OR “mRNA vaccine” OR “DNA vaccine” OR “inactivated vaccine” OR “multi-epitopes” OR “peptide vaccine” OR “pan-sarbecovirus”. The literature was searched from December 2019 to January 2023 for peer-reviewed papers reporting next-generation SARS-CoV-2 vaccines. The International Clinical Trials Registry Platform (ICTRP) was searched through http://trialsearch.who.int/ (accessed on 1 December 2022). A total of 8275 studies were identified. After excluding duplicates and irrelevant studies based on the title and abstract screening, the final number of articles included in this review was 26.

## 2. SARS-CoV-2 Variants

Ever since the detection of the first SARS-CoV-2 viral variant, it became clear that a suitable naming scheme had to be implemented to designate and keep track of novel emerging variants despite the existing nomenclatures used by the GISAID and Nextstrain. Subsequent SARS-CoV-2 variants and their lineages would be denoted by the letters of the Greek alphabet [5]. This effort was supplemented with another classification system to describe the level of severity, transmissibility and epidemiological surveillance from health authorities. These variants were labelled as Variants Under Monitoring (VUM), Variants of Interest (VOI) and Variants of Concern (VOC) (source: WHO). Throughout the COVID-19 pandemic, there were 5 VOCs reported by the WHO; *viz* B.1.1.7 (Alpha), B.1.351, (Beta), P.1 (Gamma), B.1.617.2 (Delta) and B.1.1.529 (Omicron). These variants harbored multiple mutations in the S protein which were associated with increased transmissibility, virulence and immune evasion [6].

The first VOC to be recorded, the Alpha (B.1.1.7) variant, carried the signature N501Y mutation alongside the following mutations: D614G, Δ69–70 and P681H in the S protein [7,8,9]. These mutations were linked to increased transmissibility of the SARS-CoV-2 B.1.1.7 variant, with the D614G mutation carrying high significance due to the mutation causing the viral S protein to have a stronger affinity to the target human angiotensin-converting enzyme 2 (ACE2) protein while maintaining its existing immune escape function [10,11,12]. In addition, the H69-V70 deletion modified the conformation of the NTD loop, which enhanced infectivity [7].

The Beta variant from South Africa was the first variant to display increased rates of transmission among a younger, healthier population, making it more likely that they get infected. It also caused infected individuals to be more likely to be hospitalized and increased mortality rates [13]. The signature mutations were K417N, E484K, N501Y, Δ242–244, R246I and N501Y located in the RBD. The mutations N501Y and D614G present in the Alpha variant were identified to enhance the binding affinity between the S1 subunit and the ACE2 receptor. The addition of the K417N and E484K mutations was reported to enhance the binding affinity of the spike and receptor further [14,15].

The Gamma variant, identified as P.1, emerged shortly after the Beta variant in October 2020. It was found to contain 12 spike protein mutations that conferred increased transmissibility and virulence in addition to facilitating viral escape. The mutations present in the RBD also included signature mutations such as N501Y, E484K and K417T, and these were also present in the previous Alpha and Beta variants [15].

The Delta variant (B.1.617.2) was first detected in India in late 2020. During the time frame between October and May 2021, Delta had spread to many other countries, causing subsequent waves of SARS-CoV-2 outbreaks [16]. The Delta strain showed that it had increased transmissibility and virulence when compared to the Alpha, Beta and Gamma variants. This was attributed to the mutations present in its spike protein. The mutations T478K, P681R and L452R present in Delta contributed to the increased infectivity [16].

The Omicron variant (B.1.1.529) was more contagious, with a higher transmissibility than the wild-type Wuhan strain and the Delta variant, and it has been the dominant strain of SARS-CoV-2 worldwide since December 2021. Omicron has evolved to give rise to five subvariants, *viz*. BA.1, BA.2, BA.3, BA.4 and BA.5. The high mutation rates in the spike (S) gene of Omicron subvariants (more than 30 mutations) affected the binding to ACE2 [17] and enabled them to escape from neutralizing antibodies [18,19]. The timeline of the emergence of SARS-CoV-2 variants as well as the amino acid changes present in SARS-CoV-2 variants in the spike (S) gene are summarized in Table 1.

## 3. mRNA Omicron Vaccine

Gagne et al. (2022) developed an Omicron mRNA vaccine, mRNA-1273.529, by applying a similar platform as that of the Moderna mRNA-1273 vaccine. The mRNA-1273.529 (mRNA-Omicron) vaccine encoded a full-length prefusion stabilized spike protein antigen derived from the SARS-CoV-2 Omicron variant encapsulated in lipid nanoparticles [28]. Rhesus macaques were immunized with 100 µg of mRNA-1273 vaccine at week 0 and week 4 and boosted at week 41 with 50 µg of mRNA-1274 or mRNA-Omicron. The neutralizing antibody titers against the WT strain and Omicron were higher in the sera of macaques boosted with the mRNA-Omicron vaccine; 50% inhibitory dilutions (ID_50_) were at 5360 and 2980 when compared to those boosted with the mRNA-1273 vaccine, showing ID_50_ at 2670 and 1930, respectively. The neutralizing titers against two Omicron subvariants, BA.1 and BA.2, were comparable between groups of mice that were boosted. Viral replication in the lower airways was detected following the Omicron challenge 1 month after each booster, demonstrating that boosting with the mRNA-1273 or mRNA-Omicron vaccine conferred similar protections in the lungs against Omicron [28].

## 4. mRNA Bivalent Vaccines

The FDA granted an Emergency Use Authorization (EUA) for bivalent vaccines manufactured by Moderna (mRNA -1273.222) and Pfizer-BioNTech to be administered as a single booster at least two months following primary or booster vaccination [29]. The bivalent vaccine manufactured by Pfizer-BioNTech contained 15-µg of mRNA encoding the wild-type spike protein of SARS-CoV-2, and 15-µg of mRNA from the spike protein of the Omicron BA.4/BA.5 subvariants [30]. As the spike proteins in the Omicron BA. 4 and BA. 5 variants were identical, both could be targeted with a single mRNA strand. The FDA approved the bivalent BA.4/BA.5 mRNA vaccine despite the fact that the clinical trial of Pfizer BioNTech’s bivalent BA.4/BA.5 mRNA vaccine was still ongoing (NCT05472038). The approval was based on the extensive safety and immunogenicity data from the monovalent mRNA vaccine as well as the clinical trial of the bivalent BA.1 vaccine and pre-clinical data obtained from immunization with the bivalent BA.4/BA.5 mRNA vaccine [29].

To evaluate the safety, tolerability and immunogenicity of the bivalent BA.4/BA.5 vaccine, the clinical Phase II/III trial (NCT05472038) enrolled about 900 healthy volunteers, aged 12 years and older in the United States, who had previously received at least three doses of an authorized COVID-19 vaccine [31]. Participants from 18 to 55 years of age were administered with either a 30-µg or 60-µg booster dose of the bivalent BA.4/BA.5 mRNA vaccine, while those aged 12 to 17 years old received a 30-µg booster dose of the same vaccine. Early data from a clinical trial involving 40 participants reported that the bivalent BA.4/BA.5 mRNA vaccine provided better protections against Omicron BA.4 and BA.5 subvariants than the original mRNA-based vaccine. The bivalent BA.4/BA.5 vaccine was well tolerated and had a similar safety profile to the original mRNA vaccine. Sera collected 7 days after a 30-µg booster dose of the bivalent BA.4/BA.5 vaccine showed a significant increase in the Omicron BA.4/BA.5-neutralizing antibody response above pre-booster levels [31]. Data regarding responses at one month post administration of the bivalent BA.4/BA.5 vaccine booster were still unavailable. Pfizer-BioNTech had initiated a similar Phase I/II/III trial (NCT05543616) in September 2022 to investigate the bivalent BA.4/BA.5 vaccine in children aged 6 months to 11 years of age. The Pfizer-BioNTech and Moderna bivalent vaccines were authorized by FDA for vaccination in children down to 6 months of age in December 2022 [32].

The Moderna bivalent vaccine, mRNA-1273.222, contained two mRNAs (1:1 ratio, 25 μg each) encoding the prefusion-stabilized spike glycoproteins of the original SARS-CoV-2 (Wuhan-Hu-1) and the Omicron variant BA.4/BA.5 [33]. Approval of the Moderna bivalent vaccine was based on pre-clinical findings for mRNA-1273.222 and data from the Phase II/III clinical trial of an mRNA-1273.214 bivalent booster vaccine targeting the Omicron BA.1 subvariant. A Phase II/III clinical trial for mRNA-1273.222 (NCT04927065) had fully enrolled 512 participants and was well in progress.

The neutralizing activities of the bivalent mRNA-1273.214 (1:1 mix of mRNAs encoding Wuhan-1 and BA.1 spike proteins) and mRNA-1273.222 (1:1 mix of mRNAs encoding the Wuhan-1 and BA.4/5 spike proteins) booster doses were higher than the mRNA-1273 booster [34]. Sera generated following the booster mRNA-1273.214 dose exhibited the greatest response against Omicron BA.1 (GMT: 13,183), but showed low activity against Omicron BA.4/5 (GMT: 293). The bivalent mRNA-1273.222 vaccine showed the highest neutralizing titers against BA.4/5 (GMT: 15,561). Boosting with mRNA bivalent vaccines slightly increased the protection of mice against lung pathology after intranasal challenge with Omicron BA.5 viruses [34].

Hajnik et al. (2022) developed an mRNA vaccine which encoded the full-length nucleocapsid protein of SARS-CoV-2 (Wuhan-Hu-1 strain) encapsulated in lipid nanoparticles (mRNA-N) [35]. In addition to mRNA-N, they also generated an mRNA vaccine encoding the full-length prefusion stabilized spike protein of SARS-CoV-2 (Wuhan-Hu-1 strain) with two proline mutations S-2P (mRNA-S), similar to the Pfizer BNT162b2 and Moderna mRNA-1273 vaccines. Mice or hamsters were immunized with two doses of mRNA-S or mRNA-S + N (1 µg of each mRNA) at week 0 and week 3, followed by a challenge with Delta and Omicron variants (2 × 10^4^ pfu) at week 5. When compared to mRNA-S vaccination alone, the bivalent vaccine combining both mRNA-N and mRNA-S (mRNA-S + N) was demonstrated to confer protections of both the lung and upper respiratory tract against SARS-CoV-2 Omicron and Delta challenges in hamsters. The neutralizing activities in the sera of hamsters immunized with mRNA-S + N were higher against both the WT virus (PRNT_50_: ~6000) and the Delta variant (PRNT_50_: ~1000) when compared to mRNA vaccination alone (PRNT_50 of_ WT: 2667, Delta: 440). Vaccination with mRNA-S + N also elicited robust S-specific and N-specific CD4^+^ and CD8^+^ T cell responses, as indicated by the increase in TNF-α, IFN-Ɣ and IL-2 [35].

## 5. Inactivated Omicron Vaccine

An inactivated Omicron vaccine was developed by the China National Biotec Group Company Limited and Beijing Institute of Biological Products Company. The vaccine was produced from the Omicron BA.1 subvariant (HK-OM-P0) isolated from the throat swab of a COVID-19 patient [36]. Similar to the inactivated vaccine, Sinopharm COVID-19 Vaccine (BBIBP-CorV), derived from the original SARS-CoV-2 Wuhan strain (HB02), was cultivated in Vero cells and inactivated with β-propiolactone. A two-dose immunization with middle (6 µg) and high (12 µg) doses of the Omicron inactivated vaccine promoted the production of high levels of neutralizing antibodies against the Omicron variant (BA.1) in mice. In addition, immunization with the inactivated Omicron vaccine was shown to induce a cellular immune response, as indicated by the secretion of IFN-Ɣ from T cells. The inactivated Omicron vaccine was shown to be safe and did not cause acute toxicity in rats [36]. This Omicron inactivated vaccine is currently being evaluated in Phase III clinical trial (NCT05374954) in participants aged 18 years and older with 2- or 3-dose vaccination history with the BBIBP-CorV inactivated vaccine.

## 6. DNA Vaccines

The DNA vaccine, ZyCoV-D, was developed by utilizing a pVAX-1 DNA plasmid vector to form a recombinant DNA plasmid consisting of the IgE signal sequence, followed by the S gene of the SARS-CoV2 prototype Wuhan strain. With favorable results in Phase I/II dose-escalation clinical trial (CTRI/2020/07/026352) and also in a Phase III clinical trial (CTRI/2020/07/026352) in 2021, the vaccine was shown to be safe and immunogenic, especially against the SARS-CoV-2 Delta variant [37].

The ZyCoV-D DNA vaccine developed by Zydus Cadila Healthcare in India is the only DNA vaccine against SARS-CoV-2 that has been approved by the Indian government for human immunizations. Consisting of the full-length spike protein (S) of the SARS-CoV-2 as the main antigenic region incorporated in the DNA plasmid vector pVAX1, the DNA vaccine showed promising results in preclinical and clinical stages. In the preclinical stage, intradermal administration of the DNA vaccine in various animal models such as mice, guinea pigs, and rabbits at a dose of 25, 100 and 500 µg was able to elicit humoral immune antibody responses in terms of neutralizing antibodies against SARS-CoV-2, and also elicited Th-1 response as demonstrated by a 10–12-fold increase in IFN-γ production [38].

Immunogenicity testing in phase I/II clinical trials showed both humoral and cellular immune responses [39]. Seroconversion rates at Day 56 in terms of neutralizing antibody (NAB) titers were shown to be 0%, 16·67%, 20·00%, and 10·00% in the four treatment groups of participants [1 mg, Needle; 1 mg, Needle-free injection system (NFIS); 2 mg, Needle; 2 mg, Needle-free injection system (NFIS)], respectively. Seroconversion rates were much higher at Day 84, as the NAB titers were shown to be 18·18%, 16·67%, 50·00%, and 80·00% in the four treatment groups (1 mg, Needle; 1 mg, NFIS; 2 mg, Needle; 2 mg, NFIS), respectively [39]. Intradermal administrations of 2 mg of the DNA vaccine using the needle-free injection system resulted in peak cellular response in terms of IFN-γ production, with 41.5 spot-forming cells (SFC) per million PBMCs, which lasted from Day 56 to Day 84. Similar immune responses were observed upon intradermal administrations of 1 mg of the DNA vaccine, with an IFN-γ production of 73 SFC per million PBMCs.

In Phase III clinical trial, ZyCoV-D showed an efficacy of 64·9% in mild SARS-CoV-2 infections, based on 58 of 78 mild COVID-19 infections in the placebo group and 20 mild cases in those immunized with the ZyCoV-D DNA vaccine [40]. In particular, upon administration of the vaccine, the observed antibody concentrations were significantly higher in the vaccine group (952·67 EU, 95% CI 707·94–1282·00) than those observed in the placebo group (154·82 EU, 91·25–262·70). The immunogenicity response in the group that received the DNA vaccine was shown to be higher than that of the control group (IgG seroconversion 100% versus 93·33%). Cellular responses in terms of IFN-γ response at day 56 showed a 13-fold increase in SFCs per million PBMCs, while on Day 84, the response was 9·6-fold higher in terms of production of SFCs per million PBMCs when compared with the placebo group.

Other researchers have also employed the use of advanced formulations to develop DNA vaccines against SARS-CoV-2 VOCs. One such approach was the development of a more universal DNA vaccine against SARS-CoV-2 which harbored antigenic regions from multiple SARS-CoV-2 strains [41]. More specifically, the vaccine was constructed using nucleotides encoding the receptor-binding domain, membrane, and nucleoproteins from the SARS-CoV-2 prototype Wuhan strain, as well as from the Alpha and Beta variants. The administration of the vaccine induced antibodies that could neutralize the Wuhan, Beta, and Delta strains and prevented infections from SARS-CoV-2 Wuhan, Beta, Delta, and Omicron strains. Thus, humoral and cellular immune responses induced in mice immunized with the universal DNA vaccine were able to protect against the Alpha and Beta variants as well as the Wuhan strain [41]. Indeed, the DNA vaccine was also able to induce cellular responses in terms of nucleoprotein-specific T cells and contributed to 60% of the total protection conferred as a result of the administration of the vaccine [41]. Other than demonstrating that the production of T cells was essential for the resolution of the SARS-CoV-2 infection, the data also highlighted the usefulness of developing vaccines that offered broad and functional immunity against the SARS-CoV-2 Wuhan strain and its VOCs.

Jang et al. (2022) reported the development of AcHERV-COVID19S, a human endogenous retrovirus (HERV)-enveloped recombinant baculoviral DNA vaccine against SARS-CoV-2 [42]. The researchers utilized a non-replicating recombinant baculovirus that delivered the SARS-CoV-2 S gene from the Wuhan strain. In challenge studies, the administration of the AcHERV-COVID19S DNA vaccine candidate to K18-hACE2 Tg mice conferred 50% protective efficacy upon infection with the SARS-CoV-2 Delta variant. Further development of the AcHERV-COVID19D DNA vaccine involved replacing the spike (S) protein gene from the original SARS-CoV-2 Wuhan strain with the receptor binding RBD from the S1 subunit of the Delta variant. The vaccine also contained proline substitutions and the deletion of the polybasic cleavage site to enhance immunogenicity. The cross-protection offered by the AcHERV-COVID19S DNA vaccine against infections from SARS-CoV-2 Wuhan strain and VOCs such as the Delta and Omicron strain showed that mice immunized with the AcHERV-COVID19D DNA vaccine demonstrated 100% survival when challenged with Delta and Omicron VOCs and 71.4% survival against the prototype the SARS-CoV-2 Wuhan strain [42]. The elicitation of cellular immunity was also studied by the researchers who used ELISPOT analysis to show higher levels of IFN-γ-secreting splenocytes from the spleens of C57BL/6 mice immunized with the AcHERV-COVID19S DNA vaccine as compared to naïve mice. mRNA expression levels of TNF-α, IL-2, and IL-4 were shown to be much higher in mice which had received the vaccine when compared with the control group. Immunized mice showed more potent Th1 cell immune responses and maintained a greater level of Th1 cytokine mRNA than the placebo group [42]. This data provided evidence that the goal of development of vaccines that offered cross-protection against the spread of SARS-CoV-2 VOCs could be achieved.

Recently, the development of a DNA vaccine against SARS-CoV-2 known as the pSARS2-S vaccine, which contained the S gene from the Wuhan strain and was administered through electroacupuncture in the murine model, showed that the vaccine was able to elicit high neutralizing antibody titers and IFN-γ/TNF-α-secreting CD4^+^ and CD8^+^ T cells as well as neutralizing antibodies that were able to cross-neutralize different VOCs [43]. Other approaches such as the one used by Mucker et al. (2022) employed a doggy bone DNA (dbDNA) construct which involved a novel synthetic DNA vector to develop a DNA vaccine comprising the SARS-CoV-2 spike (S) protein sequence based on the Wuhan strain. SARS-CoV-2 variants such as the Beta, Delta, and Delta^+^ VOCs when tested against sera derived from hamsters immunized with the dbDNA vaccine showed that the vaccine was able to produce cross-neutralizing antibodies against SARS-CoV-2 variants other than the Wuhan strain [44].

## 7. Protein Subunit Vaccines

V-01D-351 is a bivalent protein-based vaccine developed by Livzon Pharmaceutical Inc., China, which contains the whole RBD protein from both Beta and Delta variants (1:1 ratio), and is armed with an interferon-α at the N terminus and dimerized by human IgG1 Fc at the C terminus, as well as a pan HLA-DR binding epitope (IFN-PADRE-RBD-Fc dimer) [45]. It is currently in a Phase II clinical trial (NCT 05273528) to assess the immunogenicity and safety of the V-01-351 bivalent protein-based vaccine in adults aged 18 years and older following vaccination with two doses of the inactivated vaccines. Participants who received the V-01D-351 booster developed potent immunogenicity against the original Wuhan strain as well as substantial cross-neutralizing responses against Delta and Omicron BA.1, indicating the presence of conserved neutralizing epitopes in Beta and Delta strains with Omicron [45]. However, there were no data regarding the neutralizing capabilities against the current Omicron BA.4 and BA.5 subvariants.

Liu et al. (2022) developed a pan-sarbecovirus vaccine by fusing the RBD from the original SARS-CoV-2 strain with a Fc fragment of human IgG, and utilized a small molecule “STING agonist CF501” as the vaccine adjuvant (CF501/RBD-Fc) [46]. This vaccine was demonstrated to elicit potent cross-neutralizing antibody responses against the live original SARS-CoV-2 strain and nine pseudotyped SARS-CoV-2 variants (Alpha, Beta, Gamma, Delta, Epsilon, Zeta, Eta, Iota and Kappa), pseudotyped SARS-CoV and SARS-related coronaviruses in rabbits and rhesus macaques. Importantly, neutralizing antibodies in the sera of rhesus macaques vaccinated with two doses of the CF501/RBD-Fc vaccine could neutralize the pseudotyped Omicron variant with an NT_50_ of 6469 on day 28 after primary vaccination [47]. Three doses of the CF501/RBD-Fc vaccine in macaques generated extremely high levels of neutralizing antibodies against the pseudotyped Omicron variant, with an NT_50_ of 35,066 at day 122 following primary vaccination. Sera from the macaques were able to neutralize the authentic Omicron variant (hCoV-19/Hong Kong/HKU-344/2021) with an NT_50_ of 9322 at day 122. The levels of neutralizing antibodies against the authentic Omicron variant remained at an NT_50_ of 2430 on day 191, implying that the CF501/RBD-Fc vaccine might provide more durable protective immunity. Vaccine-induced T cell responses were assessed by determination of IFN-Ɣ elicitation by the peripheral blood mononuclear cells (PBMCs) isolated from the macaques after primary immunization using a peptide library that spanned the full-length RBD protein [46]. CF501/RBD-Fc vaccination was shown to generate strong IFN-Ɣ responses in macaques 14 days after primary vaccination, with responses remaining at a high level up to 210 days later.

## 8. Identification of Epitopes against SARS-CoV-2 Wuhan Strain and VOCs

The development of next-generation vaccines against the SARS-CoV-2 Wuhan strain and its VOCs first requires the identification of epitopes from antigenic regions that would elicit broad and long-lasting immune responses. Such an approach to develop next generation vaccines against SARS-CoV-2 is warranted because it shows promise in boosting the effectiveness of immunization and its breadth of protection against viral variants/subvariants that are constantly emerging and being transmitted to the community.

There is extensive research being conducted in identifying such immunogenic epitopes. For example, Heide et al. (2021) identified immunogenic epitopes from different structural proteins present in SARS-CoV-2 towards the generation of specific T cell epitopes [48]. These epitopes were obtained from 135 overlapping 15-mer peptides spanning the envelope (E), membrane (M) and nucleoprotein (N) of SARS-CoV-2, interacting with sera from both infected and convalescent SARS-CoV-2 patients. Peptide-specific CD4^+^ T cell responses in terms of interferon-γ (IFN-γ) production were evaluated using enzyme-linked immunosorbent spot (ELISpot) and corroborated by single-peptide intracellular cytokine staining (ICS) analysis. It was observed that 97% of the participants demonstrated the elicitation of CD4^+^ T cell responses directed towards either the N, M or E proteins. More specifically, high response frequencies were demonstrated, with a total of 10 N, M or E-specific peptides, half of these peptides showing strong binding affinities to several HLA class II binders. Notably, three peptides *viz.* Mem_P30 (aa146–160), Mem_P36 (aa176–190), and Ncl_P18 (aa86–100) were able to elicit CD4^+^ specific T cell responses in approximately 55% of participants, showing a high population coverage. While Mem_P30 and Mem_P36 belonged to the M protein, the Ncl_P18 peptide was found in the N protein. After specifying the length and HLA restriction of the peptides, a novel DRB*11 tetramer (Mem_aa145–164) was developed and used for ex vivo phenotype evaluation of SARS-CoV-2-specific CD4^+^ T cells. This in-depth analysis of single T cell peptide response showed that SARS-CoV-2 infection universally primed a broad T cell response that was focused on several specific peptides found within the N, M, and E structural proteins.

As reported by Lim et al. (2022), B cell responses could be identified by epitope identification using literature mining and bioinformatics tools to identify antigenic regions capable of eliciting humoral immune responses [49]. Although current vaccines could confer lower levels of protection against SARS-CoV-2 VOCs due to multiple mutations in antigenic regions, a satisfactory protective efficacy was still observed against SARS-CoV-2 VOCs. This protection might be attributable to cellular immunity. The identification of epitopes capable of eliciting CD8^+^ specific T cell responses is promising because multifunctional CD8^+^ T cells could allow inhibition of the viral escape of SARS-CoV-2 VOCs. The existence of conserved CD8^+^ T cell epitopes could effectively compensate for the reduction in the CD8^+^ T cell activity resulting from mutations within T cell epitopes. Boni et al. (2021) demonstrated that several immunodominant CD8^+^ T cell epitopes could be found in conserved locations within the SARS-CoV-2 genome that were highly unlikely to undergo mutations without significantly impairing functional SARS-CoV-2 genes [50]. This is particularly relevant as some immunodominant CD8^+^ T cell epitopes are located within highly conserved SARS-CoV-2 regions that could not mutate without impairing SARS-CoV-2 functionality. It was significant that several of these conserved epitopes were labelled as degenerate, which enabled them to associate with several HLA class I molecules on APCs and interact with CD8^+^ T cell populations of various HLA restrictions at the same time. Degenerate CD8^+^ T cell epitopes were seen to be logical candidates for the development of CD8^+^ T cell response-enhanced next-generation COVID-19 vaccines.

Research has also focused on identifying CD4^+^ T cell epitopes to provide protection against the SARS-CoV-2 Wuhan strain and its VOCs. Although the impact of mutations in the SARS-CoV-2 genome on CD4^+^ T cell immune responses is not well-understood, epitope mapping might provide useful knowledge regarding the ability of CD4^+^ T cells to provide broad and conserved protection against VOCs. After isolating more than 100 SARS-CoV-2-specific CD4^+^ T cell clones from recovering COVID-19 patients, Long et al. (2022) mapped HLA II restrictions of 21 epitopes on three SARS-CoV-2 proteins to evaluate the breadth of immune responses. It was observed that following vaccination, responses to the spike epitopes were also observed in people who were not infected with SARS-CoV-2 [51]. In contrast to pre-existing cross-reactive coronavirus-specific T cell responses, the absence of CD4^+^ T cell cross-reactivity with endemic beta-coronaviruses suggested that these responses were generated by naive T cells. Ten of the seventeen spike epitopes had mutations in VOCs, and seven of them, including three of the four altered in Omicron, had impaired CD4^+^ T cell recognition. This showed that the identification of broad CD4^+^ T cell epitopes might be the key to limiting immune evasion capabilities associated with SARS-CoV-2 VOCs.

The emergence of SARS-CoV-2 variants that show an increased propensity to evade antibodies has led to recurrent waves of infections with reduced vaccine efficacy. In our search for broadly protective vaccinations, there is still a crucial knowledge gap regarding the degree to which vaccine-elicited mucosal or systemic memory T cells can defend against such antibody-evasive SARS-CoV-2 variants. Using adjuvanted spike protein–based vaccines that elicited potent T cell responses, Kingstad-Bakke et al. (2022) assessed whether systemic or lung-resident CD4^+^ and CD8^+^ T cells protected against SARS-CoV-2 variants in the presence or absence of virus-neutralizing antibodies [52]. It was observed that the elicitation of mucosal response was associated with potent viral control and protection of lung pathology through the production of neutralizing antibodies. Although mucosal immunity resulted in the elicitation of mucosal memory CD8^+^ T cells, humoral immune responses had a more prominent role in being able to effectively neutralize the invading virus. In fact, mucosal memory CD8^+^ T cells were not able to confer adequate levels of protection in response to homologous SARS-CoV-2 without CD4^+^ T cells and neutralizing antibodies. Nevertheless, when virus-neutralizing antibodies were not present, memory CD4^+^ and CD8^+^ T cells were able to confer protection against the B1.351 (β) variant without symptoms of lung immunopathology. It might be useful to induce systemic and mucosal memory T cells that were directed against conserved epitopes to combat SARS-CoV-2 variants that can avoid neutralizing antibodies.

Although initial findings, which utilized in silico immunoinformatic approaches associated with vaccine development and immunogenicity, have identified these epitopes based on high levels of antigenicity and broad conservancy, future studies would need to be conducted to validate these epitopes and the resulting immune responses in both animal and human models. Nevertheless, the use of bioinformatics to predict highly conserved and immunodominant epitopes capable of eliciting potent immune responses against both the SARS-CoV-2 Wuhan strain and its VOCs serves as a useful starting point, guiding the development of next-generation vaccine platforms.

## 9. Peptide-Based Vaccines

The concept behind a SARS-CoV-2 peptide-based vaccine is to stimulate immune cells to elicit an immune response to epitopes derived from antigenic regions of the virus. However, the main advantage of this vaccine platform over the whole S-protein or nucleic acid-based vaccines is that it could trigger specific responses to the immunogenic epitopes present as peptides. Peptide vaccines could be designed to allow the induction of CD8⁺ cytotoxic T cells (CTL) that would kill infected host cells to halt the viral replication process by incorporating CD8⁺ T cell epitopes into the vaccine design. Likewise, CD4⁺ T helper cells could also be triggered by the epitopes incorporated in the peptide vaccine being designed. Alternatively, a peptide vaccine could also be designed to induce only B cell responses and the production of neutralizing antibodies which would prevent circulating viruses from infecting host cells. The rationale behind focusing on developing the peptide vaccine platform for immunizations against COVID-19 was to overcome the limitations of utilizing only the whole S protein of the Wuhan strain that was the target antigen in the first generation of COVID-19 vaccines. Currently, no peptide-based vaccines consisting of SARS-CoV-2 epitopes from the whole genome have been approved and applied clinically, but there are four vaccine candidates being developed based on the peptide-based vaccine platform that are currently in clinical trials.

1. CoVac-1

Heitmann et al. (2021) designed a vaccine centered on inducing a broad and long-lasting T cell immunity, as T cell epitopes were infrequently impacted by mutations present in variants of concern [53]. The vaccine prototype was composed of SARS-CoV-2 T cell epitopes derived from the viral spike (S), membrane (M), nucleocapsid (N), envelope (E) and open-reading Frame 8 (ORF8) proteins, combined with a Toll-like receptor 1/2 agonist XS15 emulsified in a Montanide ISA51VG adjuvant. This vaccine was designed to focus on antigenic peptides of SARS-CoV-2 that could be recognized by HLA-restricted T cells which would confer long-term immune protection. This vaccine prototype has recently undergone Phase I clinical trials (NCT4954469) involving 36 participants (18–80 years) in which the prototype was found to induce both multifunctional CD4⁺ and CD8⁺ T cell responses, targeting multiple epitopes in all the participants. Its efficacy was evaluated through comparison of IFN-γ levels from ELISPOT analysis and intracellular staining of T cells stimulated with CoVac-1 peptides against a panel of peripheral blood mononuclear cells (PBMCs) from convalescent SARS-CoV-2 patients and healthy individuals immunized with mRNA or adeno-vectored vaccines. The results reported that the immune responses mediated by CoVac-1 induced broad, potent and variant-of-concern-independent multifunctional CD4⁺ and CD8⁺ T cell activity. It was also found that the cells had a higher magnitude of induced immunity compared to those derived from natural infections or those from vaccinated individuals. No serious adverse reactions were found during the Phase I clinical trials even though local granuloma formations were observed in the participants post-immunization [53].

2. Peptide vaccine derived from epitopes from SARS-CoV-2 S and N proteins

This epitope-specifying peptide-based vaccine was developed as a proof of concept to determine if T cell mediated immunity was sufficient to confer protection from SARS-CoV-2 infection [54]. The authors also elaborated on this vaccine prototype, being focused specifically on T cell immunogenicity whereby 20 highly conserved peptides specifying epitopes derived from the S and N proteins were validated in murine models to stimulate long-lasting immunity through induction of cytotoxic CD8⁺ T cells and CD4⁺ T helper cells. All epitopes were selected with considerations of restrictions by MHC-I and MHC-II so the corresponding CD8⁺ and CD4⁺T cells would recognize the peptides and generate effective cellular immune responses by producing cytokines and activating co-stimulation signaling [54].

The emergence of VOCs such as Omicron has also prompted this shift towards alternative vaccine platforms such as multi-epitope peptide-based vaccines to elicit T cell-mediated immunity [55]. This might be a suitable solution to the resistance posed by the variants, as they are known to escape neutralizing antibody activities generated by current vaccines and even by natural immunity from past infections.

The authors selected the epitopes which were validated through the use of predictive in silico software, focusing on the target proteins for their conservancy and immunogenicity [54]. C57BL/6 mice were immunized twice at a 2-week interval with a peptide mixture or with individual peptides derived from either the S protein or N protein together with the CUK2 RNA adjuvant. The immunogenicity of the peptides was determined through expression of IFN-γ based on ICS flow cytometry, ELISpot and cytokine enzyme-linked immunosorbent analysis. The results indicated that four peptides (2 Spike and 2 Nucleocapsid) were capable of inducing the strongest T cell-mediated responses in vivo. Mice immunized with a mixture of these four peptides and the CUK2 RNA adjuvant showed increased levels of IFN-γ-producing T cells and an increased frequency of proliferating T cells. However, there was a slight reduction in viral titers, and virus-induced injury was observed in the lungs after SARS-CoV-2 challenge. This finding indicated that the peptides might not be able to elicit immune responses to completely eliminate the virus. Currently, this vaccine candidate has only been tested in animals and there is no information regarding the efficacy of the vaccine in humans; however, the authors maintain that they are aiming to continue the development of a peptide-based vaccine based on the peptides that were validated in the murine model.

3. UB-612

UB-612 is a multi-epitope peptide-based vaccine candidate developed by Vaxxinity, USA. The UB-612 vaccine construct contained an RBD (derived from the WT Wuhan strain) which was fused to a modified single-chain human IgG1 Fc protein (S1-RBD-sFc), a UBITh1a peptide as a catalyst for T cell activation, and a mixture of five synthetic Th/CTL peptides derived from the spike protein, nucleocapsid and membrane proteins produced in CHO cells [56]. Synthetic peptides were stabilized with a negatively charged oligonucleotide (CpG1) and adsorbed on aluminum phosphate adjuvant. These peptides were found to be highly conserved across all VOCs, including Delta and Omicron variants, and were able to bind to human MHC I and II with broad HLA genetic coverage as well as induction of T cell proliferations.

UB-612 was shown to be safe and well tolerated in a Phase I/II clinical study, and it was shown to generate a lasting neutralizing antibody response with a half-life of 187 days and a sustained T cell response in individuals aged 20 to 55 (NCT04545749 and NCT04773067) [57]. Three doses of UB-612 induced significant neutralizing antibody titers against live Delta variants (VNT_50_ of 2358) when compared to live WT virus (VNT_50_ of 3992) in a Phase I study with a 100-µg booster (NCT04967742), and neutralizing titers showed only a small 1.7-fold reduction [57]. The UB-612 vaccine was found to have cross-reactive neutralizing antibody titers against pseudotyped SARS-CoV-2 variants such as Alpha, Beta, Gamma and Omicron (BA.1), when compared to the WT pseudovirus (pVNT_50_ of Alpha: 9300, Beta: 4974, Gamma: 13408, Omicron: 2325 versus WT: 12778). A third dose of the UB-612 vaccine administered 7–9 months after the primary vaccination dramatically boosted neutralizing antibody titers against Omicron and the WT strain to VNT_50_ of 670 and 970, respectively, with only a small 1.4-fold reduction against the Omicron variant when compared to the 5.5-fold reductions observed in the pseudovirus assay [57,58].

The UB-612 vaccine is currently being assessed in a Phase III clinical study as a booster vaccine for vaccinees who have received primary immunizations with mRNA (Pfizer), adenovirus vectored (AstraZeneca), or inactivated virus (Sinopharm BIBP) vaccines (NCT05293665). Three doses of UB-612 at 100 µg per dose elicited neutralizing antibody titers with geometric mean titer (GMT) virus neutralization titer (VNT_50_) at 335 against the Omicron variant, which was more than 3-fold higher than the neutralizing antibody titers observed following three doses of the Pfizer mRNA vaccine [59].

4. EpiVacCorona

This vaccine candidate was designed based on the immunodominant epitopes being presented as peptides that represented the antigenic regions [60]. These peptides were selected through computational bioinformatics with reference to X-ray diffraction analysis data of homologous SARS-CoV spike protein and the SARS-CoV-2 genetic sequence. Only epitopes that were located near the S1 and S2 sites that were vital to the virus were selected for the vaccine candidate. Computational analysis was also used to exclude potentially toxic peptides. A carrier protein derived from the SARS-CoV-2 N protein was used as a substrate onto which the selected epitopes were covalently bound. The final vaccine candidate was formulated with peptide immunogens of the S protein of the SARS-CoV-2 conjugated to a carrier protein, adjuvanted with aluminum hydroxide and administered through intramuscular injections.

Phase I/II (NCT04527575) and Phase III/IV (NCT04780035) clinical trials of EpiVacCorona were performed in Russia in November 2020 and March 2021, respectively. In the first trial, participants were divided into a non-randomized trial (*n* = 14; Group 1) and a single-blind, placebo-controlled randomized trial (two groups: Group 2, *n* = 43; Group 3, *n* = 43). The volunteers in Group 1 received two doses of EpiVacCorona intramuscularly on Day 0 and Day 21. The participants were monitored for up to five days after each immunization for adverse reactions with standard physical, biochemical, immunological and haematological blood examinations. Following the monitoring period, with no adverse symptoms being observed, evaluation of EpiVacCorona was continued for the randomized trial whereby Group 2 participants receiving the immunization of EpiVacCorona were compared with the participants in Group 3 receiving only the placebo.

Based on the findings from each group, the vaccinees only reported mild local reactions after two immunizations. The vaccine was shown to be of low reactogenicity and was safe for use in immunizations. The most common adverse reaction was local pain at the injection site (observed in four out of 43 volunteers receiving the EpiVacCorona after the first dose and in two more patients after the second dose). All local reactions were mild and transient, lasting 1–2 days. There was one incident where a participant experienced a rise in body temperature 12 h after the first injection. However, three cases of acute respiratory viral infection (ARVI) occurred during the study, which were later confirmed to be due to COVID-19 infections.

The immunogenicity of the vaccine was evaluated by ELISA and neutralization titers. The vaccine induced IgG seroconversion by Day 42 after the first dose in 82% of the participants who had received EpiVacCorona. Neutralization assays performed in Vero E6 cells using diluted sera at 1:160 showed that all the participants had demonstrated neutralizing antibodies by Day 42 after the first immunization. The participants receiving the placebo did not show any seroconversion [60].

Phase III clinical trials were also performed in the same year, with over 3000 volunteers enrolling in the study, but the results were not made public [61]. Despite the lack of effective protection against the SARS-CoV-2 Delta strain, the vaccine had been registered with the Russian government and given emergency authorization for immunizations domestically.

## 10. Vectored-Based Vaccines

The majority of COVID-19 vaccinations in use are delivered intramuscularly. There are also other promising vaccination approaches such as aerosol inhalation and intranasal administrations, which could generate a mucosal immune response directly at the point of virus entry in the respiratory system. For example, two doses of aerosolized adenovirus type-5 vector-based COVID-19 vaccine (Ad5-nCov) were demonstrated to induce neutralizing antibodies and T cell responses in a Phase I clinical trial involving 130 participants [62]. However, the durability of antibodies and cellular responses as well as the cross-neutralizing activity of this vaccine against SARS-CoV-2 variants were not reported.

A single dose of intramuscular vaccination of an adeno-associated vector vaccine (AAVCOVID-1), a spike gene-based vaccine candidate encoding the S gene of the Wuhan strain, was shown to protect nonhuman primates from SARS-CoV-2 challenge after a single low dose (10^10^ genome copies) [63]. Most of the currently approved vaccines except for CanSino adenovirus type 5 (AD5)-vectored vaccine (Ad5-nCoV) or the adenovirus type 26 vectored vaccine Ad26.COV2.S (Johnson & Johnson), require two doses for full protection [64,65]. The neutralizing activities and T cell responses elicited by the AAVCOVID-1 vaccine were potent and durable for up to 11 months [63]. Importantly, the immune sera from nonhuman primates vaccinated with AAVCOVID-1 showed some degree of neutralization against all four SARS-CoV-2 variants (Alpha, Beta, Gamma and Delta) in a pseudovirus-based neutralization assay on week 14.

Modified vaccinia virus Ankara (MVA) is a highly attenuated virus strain having a replication defect in mammalian hosts [66]. MVA-SARS-2-S is a vectored vaccine based on the modified vaccinia virus Ankara that expressed the full-length SARS-CoV-2 spike (S) protein. The MVA vaccine was shown to induce SARS-CoV-2-specific T cell responses and neutralizing antibodies as well as protection of vaccinated mice from lung infections after a SARS-CoV-2 challenge. However, the durability of the cellular and humoral responses as well as the cross-neutralizing antibody responses against SARS-CoV-2 variants were not evaluated.

## 11. Live Attenuated Vaccines

COVI-VAC is a live attenuated vaccine candidate created by recoding of the SARS-CoV-2 WT (Wuhan strain) using the synthetic attenuated virus engineering (SAVE) strategy of codon pair bias deoptimization (CPD) [67]. The recoding of the SARS-CoV-2 genome in the COVI-VAC vaccine candidate resulted in 283 silent point mutations in the gene encoding the spike (S) protein [68]. The furin cleavage site in the spike (S) protein of COVI-VAC was also deleted to increase its safety. A single intranasal administration of COVI-VAC in Syrian golden hamsters elicited neutralizing antibodies that were as effective as the WT virus and conferred protection against WT challenge by reducing viral loads in the lung and brain [68].

cCPD9 is a live attenuated vaccine generated by genetically modifying the SARS-CoV-2 genome using codon pair deoptimization (CPD). The attenuated vaccine candidate, cCPD9, was produced by recoding nine fragments of the SARS-CoV-2 genome introduced by the reverse genetics system. A single dose of intranasal immunization with cCPD9 elicited strong neutralizing antibody responses and provided complete protection against SARS-CoV-2 challenge in hamsters [69].

However, there was no assessment of T cell responses and the durability of protective immunity for both the COVI-VAC and cCPD9 vaccine candidates. Live attenuated vaccines are considered to be the most effective vaccines because they generally elicit broad, robust and long-lasting immune responses similar to those induced by natural infections caused by the WT strain [70]. However, the safety, immunogenicity and efficacy of both live attenuated vaccine candidates should be further investigated in non-human primates and humans. The main disadvantage of live attenuated vaccines is the risk of reversion to virulence. Both COVI-VAC and cCPD9 were expected to be genetically stable due to the large number of mutations in the genome.

Another important advantage of live attenuated vaccines is that they can trigger immune responses, not only against the spike protein, but against the entire ensemble of viral proteins. Therefore, live attenuated vaccinations should be effective against all SARS-CoV-2 variants. A single intranasal droplet vaccination with sCDP9 was demonstrated to elicit strong cross-neutralizing antibody responses against the four SARS-CoV-2 variants (Alpha, Beta, Gamma and Delta) [71], whereas COVI-VAC vaccination conferred protection against heterologous challenge with the SARS-CoV-2 Beta variant (B. 1.351) in the Syrian Golden hamster model [72].

## 12. Discussion

Although mRNA vaccines have shown the highest efficacy and safety among the licensed COVID-19 vaccines, the disadvantages of mRNA vaccines are their high cost and low stability with short half-life. The mRNA vaccines also require extremely low temperatures (−80 °C) for storage and transportation which make them unaffordable and logistically impractical for many low-income countries. We have summarized the advantages and disadvantages of each vaccine platform in Table 2. The production cost for the adenovirus vectored vaccine was lower, at $ 0.15/dose, while Pfizer and Moderna mRNA vaccines could range from $ 14.70 to $ 23.50/dose, and the DNA vaccine was priced at $ 3.53/dose [73,74].

The development of next-generation DNA vaccines is promising and is geared towards preventing SARS-CoV-2 VOC infections. DNA vaccines that do need extremely low temperature for storage and transportation are preferable for large-scale immunizations in underdeveloped countries that do not have sophisticated infrastructures. However, due to the small amount of antigens that could transfect target APCs in vivo, the immunogenicity of DNA vaccines must be improved by incorporating chemical, genetic, and molecular adjuvants as well as considering alternative delivery systems such as electroporation and needle-free intradermal patches to increase transfection efficiencies and the elicitation of potent immune responses.

Since the DNA vaccine platform is easily modifiable and offers convenience in plasmid design, feasible production for large-scale immunizations, and easy storage at room temperature, it has received considerable attention as a promising vaccine platform against SARS-CoV-2 and its VOCs [37]. However, DNA vaccines need to address two main concerns which involve degradation by host nucleases as well as a low APC transfection efficiency [75,76]. Nevertheless, the use of gene guns, electroporations and needle-free injections was shown to successfully address these problems to elicit potent immune responses in the clinical trials of the current DNA vaccines against SARS-CoV-2 [37]. For example, a sustained humoral response was reported following the administration of the second dose of SARS-CoV-2 DNA vaccine INO-4800. Homologous booster doses were seen to significantly augment the immune response. Increasing the dose of the vaccine from 1 mg to 2 mg was reported to significantly increase the number of cytokine producing T cells and activated CD8^+^ T cells with lytic potential [77]. Moreover, the use of a needle-free injection system in Phase III clinical evaluation of ZyCoV-D (CTRI/2020/07/026352) showed that the administration of the DNA vaccine elicited potent immune responses, as seen from high seroconversion rates, neutralizing antibody titers and elevated IFN-γ levels compared to the placebo treatment [40].

An important issue associated with preventing the spread of infections resulting from the SARS-CoV-2 Wuhan strain and VOCs is vaccine hesitancy, which poses a global health challenge that significantly affects the spread of infections in pandemics [78]. Vaccine hesitancy and the failure to achieve good herd immunity are especially harmful for high-risk groups, such as older individuals and those with adverse health conditions [79]. The occurrence of complications such as vaccine-induced immune thrombotic thrombocytopenia (VITT) is a major contributing factor which has great potential to exacerbate vaccine hesitancy. To date, VITT has been associated only with the adenoviral vector-based platform against SARS-CoV-2. More specifically, five cases of VITT were reported among 130,000 vaccinees immunized with the ChAdOx1 CoV-19 vaccine manufactured by AstraZeneca [80]. Another vaccination with AS26.COV2.S produced by Johnson & Johnson reported 3.8 VITT cases per million vaccinees (approximately 1 in 263,000) [81]. In contrast, mRNA vaccines were not associated with such concerns of VITT. The self-adjuvating activity of the lipid nanoparticle mRNA-formulated vaccine was also reported to elicit potent antigen-specific cellular and humoral immune responses. Another disadvantage associated with the use of adenoviral vector-based vaccines is the induction of an immune response against the adenoviral vector itself [82].

The only approved DNA plasmid-based COVID-19 vaccine, the ZyCoV-D vaccine, has not had any reports of the occurrence of VITT upon administration in immunized individuals. DNA vaccines are associated with the risk of possible insertional mutagenesis. However, over the years and during the COVID-era, testing of DNA vaccines in preclinical trials did not show any insertional mutagenesis activity in terms of integration into the host genome [83].

For the elicited immune response to be sustained for longer periods of time, prime-boost regimens could be adopted. Traditionally, prime regimens were homologous, such that the same vaccine was used for priming and boosting. However, a newer, promising strategy is the combined use of a mix-and-match vaccine immunization approach which utilizes different platforms to deliver the same antigen in prime-boost vaccinations. Alternatively, a heterologous prime-boost immunization approach could be adopted, using different antigens or different platforms.

The development of peptide-based vaccines for SARS-CoV-2 that incorporate antigenic structures that are easily recognizable by immune cells and could trigger rapid immune responses to the emergence of VOCs is desirable [84,85]. At the start of the pandemic, there was a motivation to develop vaccines that would protect against infection; however, the emergence of the VOCs that could resist neutralizing antibody responses triggered by the Pfizer, Moderna and AstraZeneca vaccines compromised this strategy [86,87,88]. Thus, peptide vaccines that could be designed to target very specific antigens and induce immunogenicity should be considered. The clinical trials for the peptide-based vaccines described in this review also showed that the development of this particular platform is progressing at an acceptable pace.

The neutralizing activities and T cell responses elicited by the adeno-associated vectored vaccine (AAVCOVID-1) were potent and durable for up to 11 months [63]. Three doses of CF501/RBD-Fc vaccinations elicited neutralizing antibodies maintained at extremely high levels in macaques for as long as 191 days (approximately 6 months). The T cell response in macaques immunized with CF501/RBD-Fc remained high at day 210 post-first immunization [46]. The available data on the durability of other vaccines were limited. Two doses of the Moderna mRNA vaccine were shown to elicit antibodies that could last for at least 6 months [89].

Development of pan-sarbecovirus or pan-β-CoV vaccines against emerging SARS-CoV-2 variants could be the best approach to developing SARS-CoV-2 vaccines [90]. A pan-sarbecovirus vaccine (CF501/RBD-Fc) was demonstrated to elicit potent cross neutralizing antibody responses against the original SARS-CoV-2 strain, nine SARS-CoV-2 variants (Alpha, Beta, Gamma, Delta, Epsilon, Zeta, Eta, Iota and Kappa), and pseudotyped SARS-CoV and SARS-related coronaviruses in rabbits and rhesus macaques [46].

Studies have reported that mucosal immune responses prevented SARS-CoV-2 replication at the entry point and reduced viral transmission [91,92]. Secretory IgA antibodies played a critical role in defense against infection at mucosal sites. However, the data on mucosal immunity for each vaccine were limited. Boosting with either the mRNA-1273 or mRNA Omicron vaccine was shown to enhance mucosal IgG antibody and neutralizing responses to WT, Beta, Delta and Omicron, with GMTs of ~10^12^ for WT, Beta and Delta and ~10^10^ for Omicron [28]. Although intramuscular vaccination with Ad5-nCoV elicited higher concentrations of RBD-binding IgA (GMT: 521 EU/mL) than the aerosolized Ad5-nCoV (GMT: 148 EU/mL) at day 28 after the first vaccination, mixed vaccinations (an intramuscular vaccination of Ad5-nCoV followed by an aerosolized Ad5-nCov booster) elicited significant levels of IgA, with a GMT of 777 EU/mL at day 28 after a booster involving aerosol vaccination [62]. A single dose of AAVCOVID vaccination elicited a detectable level of RBD-binding IgA in the bronchoalveolar lavage harvested at 5 months following immunization [63].

## 13. Conclusions

The COVID-19 pandemic has entered its fourth year, and there are no signs of it slowing down. The SARS-CoV-2 virus is predicted to become endemic in countries such as Israel [93]. Infectious respiratory diseases such as influenza have become endemic due to their ability to cause reinfections despite the existence of vaccines against multiple influenza serotypes and the efforts involved in vaccinating populations globally. Through re-examining the patterns of influenza infections and comparing them with SARS-CoV-2, the likelihood of achieving complete elimination of SARS-CoV-2 infections through herd immunity from vaccinations has been proven insignificant [94].

The likelihood of achieving herd immunity against SARS-CoV-2 appeared to be low due to the following reasons. Firstly, there were reports based on longitudinal observations that demonstrated that humoral responses declined over time following immunizations with currently approved vaccines against SARS-CoV-2 [95]. For example, while immunization with the BNT162b2 Pfizer vaccine did lead to peak elevated levels of antibodies at weeks 4 and 5 post immunization, these titers declined shortly after. The second dose also led to elevated antibody levels, but these also decreased significantly, especially in older vaccinees [96]. Moreover, vaccine hesitancy has presented itself as a major obstacle. There were reports of the occurrence of VITT in those immunized with the adenoviral vectored-based ChAdOx1 vaccine, as well as concerns about antibody-dependent enhancement (ADE) leading to adverse effects of the mRNA vaccines. The goal of achieving herd immunity is further hampered by the emergence of new variants with immune evasion capabilities. The Omicron VOC could evade neutralization by antibodies produced in vaccinees who had received one or two doses of the vaccine, particularly when antibody titers were declining. Three doses of the spike-based vaccine may only partially protect against SARS-CoV-2 WT infection.

However, the development of next-generation vaccines capable of providing broad protection against the SARS-CoV-2 Wuhan strain and VOCs presents a promising strategy. This would involve the use of reverse vaccinology approaches that utilize in silico immunoinformatic approaches to identify highly conserved epitopes, which could be validated for their potent immunogenicity through immunizations in mice or non-human primates.

Accelerated developments in next-generation vaccine platforms showed that recombinant protein, mRNA, and DNA vaccines have advantages over traditional LAVs and IVs that utilize whole viruses. mRNA vaccines have demonstrated highly immunogenic responses that provide a high level (>94%) of immune protection against symptomatic and severe infections prior to the emergence of the Omicron variant. The mRNA platform as well as the DNA platform would allow newly validated epitope sequences to be easily incorporated through the inclusion of new gene sequences. These newly modified mRNA or DNA vaccines could be considered for development as vaccine booster doses to prevent re-infections by the WT (Wuhan) strain or by VOCs. Since mRNA vaccines require strong logistics to control their transport and storage requirements, which might hamper their usefulness and availability, DNA vaccines could offer convenience in storage and accessibility for developing countries that lack sufficient infrastructure. While recombinant protein vaccines might require extensive purification steps, they have been shown to elicit potent immune responses upon administration, and the technology of production is more amenable to low-income countries. The development of recombinant protein or peptide-based COVID-19 vaccines might set the stage for further enhancement of this platform, as the antigens encoded by the plasmid would not contain potentially harmful components, thus maintaining a stellar safety profile [97]. This trait of being able to modify the design of the DNA or recombinant peptide vaccines is of particular interest due to the benefits it entails in terms of rapidly emerging VOCs that necessitate accelerated modifications to existing antigenic sequences included in currently approved vaccines. This indicated that the antigens, once validated, might be readily incorporated in multi-epitope recombinant protein, mRNA or DNA vaccines.

## Figures and Tables

**Table 1:** Timeline of emergence of SARS-CoV-2 variants and mutations present in Alpha. Beta, Gamma, Delta and Omicron variants in the viral spike (S) gene.

Timeline of Emergence of SARS-CoV-2 Variants				
**September 2020**	September 2020	October 2020	November 2020	November 2021
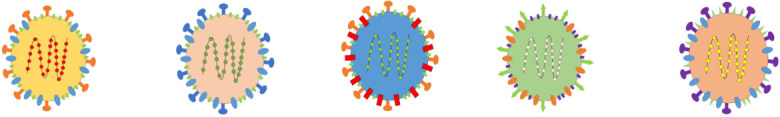				
**Alpha (B.1.1.7)**	**Beta (B.1.351)**	**Gamma (P.1)**	**Delta (B.1.617.2)**	**Omicron (B.1.1.529)**
**Mutations:** Δ69-70, Δ144, N501Y, A570D, D614G, P681H, T716I, S982A, D1118H **Pathological effects:** i. Increased transmissibility and rate of viral replication [7] [Meng et al., 2021] ii. Increased binding affinity between RBD region and hACE-2 receptor [20,21] [Zhang et al., 2020; Harvey et al., 2020]	**Mutations:** L18F, D80A, D215G, Δ242-243, K417N, E484K, N501Y, D614G. A701V **Pathological effects:** i. Reduced protective effects of existing vaccines and monoclonal antibodies [20,22] [Zhang et al., 2020; Wibmer et al., 2021] ii. Evasion of immune responses and increased transmissibility [20,21,23] [Zhang et al., 2020; Harvey et al., 2020; Tegally et al., 2020]	**Mutations:** L18F, T20N, P26S, D138Y, R190S, K417T, E484K, N501Y, H655Y, T1027I **Pathological effects:** i. Neutralizing antibodies had reduced effectiveness due to loss of strong binding affinity [20] [Zhang et al., 2020] ii. Enhanced viral entry pathways through endosomal uptake [21] [Harvey et al., 2020]	**Mutations:** T19R, G142D, E156G, Δ157-158, L452R, T478K, D614G, P681R, D950N **Pathological effects:** i. Increased viral transmissions and virulence alongside enhanced immune escape [24,25] [Di Giarcomo et al., 2021; Tchesnokova et al., 2021] ii. Infections were more likely to result in hospitalizations and mechanical ventilation [24,25] [Di Giarcomo et al., 2021; Tchesnokova et al., 2021] iii. Evasion of immune responses [26] [Liu et al., 2021]	**Mutations:** G142D, G339D, S373P, S375F, K417N, N440K, S477N, T478K, E484A, Q493R, Q498R, N501Y, Y505H, D614G, H655Y, N679K, P681H, N764K, D796Y, Q954H, N969K **Pathological effects:** i. Mutations increased binding affinity of virus to host cells [27] [Shah et al., 2021] ii. Novel mutations present in the RBD increased transmissibility [27] [Shah et al., 2021]

**Table 2:** Advantages and disadvantages of different SARS-CoV-2 vaccine platforms.

Vaccine Platforms	Live Attenuated Vaccine	Inactivated Vaccine	mRNA Vaccine	DNA Vaccine	Peptide-Based Vaccine	Adenovirus Vectored Vaccine	Recombinant S Protein
**Advantages**	Simple production, easy storage, distribution and administration. Only one dose is required. Trigger immune responses against the whole virus. Considered the most effective vaccine it can elicit robust and long-lasting humoral and cellular immune responses.	Very safe because the virus is killed and no serious adverse effects. Easy for transport and storage.	Easy and quick to design. Large-scale production is feasible. Safe as no infectious virus handling is required. Can induce humoral and cellular responses.	Low cost of production. Safe and well-tolerated. Stable under room temperature at 2–8 °C Highly adaptable to the incorporation of DNA sequences of newly emerging variants of concern.	No risk of infection. Induce specific immune responses with minimal allergic and toxic properties. Highly conserved B cell epitopes can elicit cross-reactive antibodies. Highly conserved CD4^+^ and CD8^+^ T cell epitopes can confer broad protection. Can be incorporated in expression plasmids to produce recombinant peptides	Low cost of production. Good stability at 2–8 °C. Replication-defective vectored viruses tend to elicit stronger immune responses than killed viruses. Can induce humoral and cellular responses with a single dose of Ad5-nCoV or Ad26.COV2.S.	Focus on the most immunogenic S protein of the virus for protection. The most immunogenic vaccine platform. Incapable of causing infections.
**Disadvantages**	Risk of reversion to virulence.	Significant risk due to the growth of large amounts of live viruses before inactivation. The inactivation process might affect antigen immunogenicity. Adjuvants are required. Multiple doses are needed every 12 months.	High cost. mRNA vaccines exhibit instability due to liposome formulation and require storage at −80 °C. Unaffordable and logistically impractical for many low-income countries.	Lower immunogenicity. Risk of genomic integration. Require adjuvants to enhance immunogenicity. Administration requires a medical device such as an electroporator. Needless patch is still under development.	Require peptide synthesis chemically. An adjuvant maybe needed to boost immunogenicity. Lower immunogenicity than live attenuated vaccine and mRNA vaccine.	Pre-existing immunity against viral vectors can attenuate immune responses. Some candidates require storage at −20 °C. May trigger rare but serious side effects of vaccine-induced thrombotic thrombocytopenia (VITT) and blood clots.	Require multiple purification steps involving column chromatography. An adjuvant may be needed to boost long-term immunity.

## Data Availability

Google Scholar (https://scholar.google.com/) and PubMed (https://pubmed.ncbi.nlm.nih.gov/) databases were used to perform literature search. The International Clinical Trials Registry Platform (ICTRP) was searched through http://trialsearch.who.int/.
